# Retrospective Analysis of Treatment Workflow in Frame-Based and Frameless Gamma Knife Radiosurgery

**DOI:** 10.7759/cureus.28606

**Published:** 2022-08-30

**Authors:** Jian Liu, Anuj Goenka, Emel Calugaru, Jameson Baker, Yijian Cao, Michael Schulder, Jenghwa Chang

**Affiliations:** 1 Brown University Radiation Oncology, Rhode Island Hospital, Providence, USA; 2 Radiation Medicine, Northwell Health, Lake Success, USA; 3 Department of Radiation Medicine, Donald and Barbara Zucker School of Medicine at Hofstra/Northwell, Hempstead, USA; 4 Department of Neurosurgery, Zucker School of Medicine at Hofstra, Manhasset, USA; 5 Department of Physics and Astronomy, Hofstra University, New York, USA

**Keywords:** gamma knife, patient in-clinic time, workflow, frameless treatments, frame-based treatments

## Abstract

Objective

To improve the efficiency of frame-based and frameless Gamma Knife® Icon™ (GKI) treatments by analyzing the workflows of both treatment approaches and identifying steps that lead to prolonged patient in-clinic or treatment time.

Methods

The treatment processes of 57 GKI patients, 16 frame-based and 41 frameless cases were recorded and analyzed. For frame-based treatments, time points were recorded for various steps in the process, including check-in, magnetic resonance imaging (MRI) completion, plan approval, and treatment start/end times. The time required for completing each step was calculated and investigated. For frameless treatments, the actual and planned treatment times were compared to evaluate the patient tolerance of the treatment. In addition, the time spent on room cleaning and preparation between treatments was also recorded and analyzed.

Results

For frame-based cases, the average in-clinic time was 6.3 hours (ranging from 4 to 8.7 hours). The average time from patient check-in to plan approval was 4.2 hours (ranging from 2.8 to 5.5 hours), during which the frame was placed, stereotactic reference MRI images were taken, target volumes were contoured, and the treatment plan was developed and second-checked. For patients immobilized with a mask, treatment pauses triggered by the intra-fractional motion monitoring system resulted in a significantly longer actual treatment time than the planned time. In 50 (or 55%) of the 91 frameless treatments, the patient on-table time was longer than the planned treatment time by more than 10 minutes, and in 19 (or 21%) of the treatments the time difference was larger than 20 minutes. Major treatment interruptions, defined as pauses leading to a longer than 10-minute delay, were more commonly encountered in patients with a planned treatment time longer than 40 minutes, which accounted for 64% of the recorded major interruptions.

Conclusion

For frame-based cases, the multiple pretreatment steps (from patient check-in to plan approval) in the workflow were time-consuming and resulted in prolonged patient in-clinic time. These pretreatment steps may be shortened by performing some of these steps before the treatment day, e.g., pre-planning the treatment using diagnostic MRI scans acquired a few days earlier. For frameless patients, we found that a longer planned treatment time is associated with a higher chance of treatment interruption. For patients with a long treatment time, a planned break or consideration of fractionated treatments (i.e., 3 to 5 fractionated stereotactic radiosurgery) may optimize the workflow and improve patient satisfaction.

## Introduction

For over 50 years, Gamma Knife® (GK; Elekta AB, Stockholm, Sweden) has been used in single-fraction stereotactic radiosurgery (SRS) for treating patients with benign and malignant lesions as well as functional disorders of the brain [1]. Previous generations of GK units exclusively employed a frame-based system for patient immobilization and stereotactic localization [2]. Gamma Knife Icon™ (GKI), the newest generation of GK unit introduced in 2015, is equipped with a cone-beam computed tomography (CBCT) system, which allows stereotactic localization of patients immobilized using a frameless approach, i.e., a thermoplastic mask [3,4]. Frameless immobilization avoids the trauma from the placement of a traditional stereotactic frame and is ideal for the delivery of multifraction SRS [5,6].

In frame-based GK treatment, the patient’s head is secured to a metal frame using four screws. The frame serves two purposes: immobilization and stereotactic localization. In frameless treatment, the patient’s head is fixed to the treatment couch using a thermoplastic mask. The onboard CBCT imager takes a CBCT image before treatment as the stereotactic reference. During treatment, the intra-fractional motion of the patient's head is monitored by a high-definition motion management (HDMM) system that utilizes two infrared stereo cameras to monitor the relative movement of a reflective sticker placed on the patient nose to the stickers placed on the frame where the patient head rests. When the relative movement is greater than a predefined threshold value (e.g., 1.5 mm, the default value) for 2 seconds, the sources will retract to their temporary storage position. If the relative movement remains greater than the threshold value for more than 30 seconds, the treatment couch will retract to its home position. If the patient cannot be coached back to a position within the tolerance, another CBCT will have to be taken to re-localize the patient.

The workflows for the frame-based and frameless immobilization techniques are quite different [1]. In our institution, all treatment steps for frame-based cases, including frame placement, imaging, and treatment planning and delivery, are completed on the same day in a single treatment. The frameless workflow, on the other hand, allows mask fabrication, imaging, and treatment planning a day prior to treatment, as well as both single-fraction and fractionated treatments.

It is difficult to predict the treatment time for both frame-based and frameless GK treatments, which presents a challenge for patient scheduling and often leads to long patient waiting times. For frame-based cases, the planned treatment time depends on parameters such as the number, size, location, and shape of lesions, most of which are not completely known until the day of treatment. For frameless cases, these planning parameters can be known in advance and the treatment time estimated from preplanning if the imaging is done a day before. However, the mask does not provide the same degree of immobilization as the frame. If a patient moves out of the tolerance, treatment will be paused and may even require another CBCT to re-localize the target before treatment can be resumed [7]. For certain patients, treatment interruptions can significantly prolong the treatment time.

In this study, the workflows of frame-based and frameless GKI treatments at our institution were recorded and analyzed. Factors that affect patient treatment efficiency were identified. Suggestions to improve workflow efficiency and patient satisfaction were proposed.

## Materials and methods

Gamma Knife Icon workflow

Both single and multi-fraction GK SRS with framed or frameless immobilization are performed at our clinic. Details on how the attending radiation oncologist determines the fractionation scheme and immobilization technique are beyond the scope of this study and will only be briefly described here. In short, the primary consideration is a clinical necessity. For example, three or five-fraction treatments are usually chosen for a larger treatment volume to reduce normal tissue complications. For single-fraction treatments, both framed and frameless immobilizations were used, depending on the preference of patients and/or attendings. The global impacts on patients and on hospital resources due to multi-fraction treatments were not a concern since the patient load of our GK program didn’t reach its full capacity during the study period between September 10, 2019, and November 19, 2019. In addition, our clinic is an outpatient facility, so the impact on patients is the same as those outpatient facilities performing radiosurgery using other delivery modalities (e.g., linear accelerator).

For readers who are not familiar with the workflow of external beam radiotherapy, Guo et al. have published a general framework of various time points for external beam radiotherapy, particularly for linear accelerator-based treatments [8]. The GK workflow is similar to but not exactly the same as that of the linear accelerator. Further on, we define the steps of GK workflows at our clinic, which correspond to the T2-T5 steps listed in the study by Guo et al. [8].

At our institution, a typical workflow for frame-based patients consists of six steps: (1) On the day of treatment, the patient arrives and, following completion of the appropriate paperwork, IV placement, and premedication, has the frame placed by the neurosurgeon; (2) The patient is sent to the radiology department for magnetic resonance imaging (MRI); (3) When the MRI images are imported into the GK planning system, the radiation oncologist and/or the neurosurgeon contours the target(s); (4) A treatment plan is developed, reviewed, and second-checked; (5) Once all involved parties agree on a plan, the plan is approved and sent to the GK control console for delivery; (6) The therapist takes the patient into the treatment room when the treatment room is available and ready, sets up the patient, and delivers the planned treatment.

The workflow of a frameless treatment consists of similar steps listed above but there are some major differences. First, there is no frame placement in Step (1). Instead, a thermoplastic mask is fabricated for patient immobilization and a CBCT scan is acquired for stereotactic localization. In addition, the intra-fractional patient motion is monitored during the treatment in Step (6), and the treatment might be interrupted if the monitored patient motion is above a preset threshold. At our institution, the default value of 1.5 mm is used as the tolerance of intra-fractional movement for frameless treatments. Finally, all steps other than step (6) are not required to either happen on the treatment day or follow the exact numerical order. That is, only step (6) takes place on the treatment day and the other five steps can be performed on various days earlier.

Usually, a T1-weighted magnetization-prepared rapid acquisition with gradient echo (MP-RAGE) scan is taken for MRI, which takes about 30 minutes. A T2 fluid-attenuated inversion recovery (FLAIR) MRI scan might also be acquired if the neurosurgeon or radiation oncologist orders it. The FLARE scan takes about 15 minutes. For frameless cases, MRI and simulation (including mask fabrication and CBCT reference scan) are typically completed prior to the treatment day.

Frame-based treatments at our institution are typically scheduled on Tuesday while frameless cases could be on any day from Monday to Thursday. If there is more than one frame-based treatment on the same day, these patients are scheduled to arrive at least one hour apart so that later patients do not have to wait while the neurosurgeon is placing the frame on the earlier patient.

Patient demographics

Treatment processes for 57 (16 frame-based and 41 frameless) GKI patients between September 10, 2019, and November 19, 2019, were followed, recorded, and analyzed. Leksell GammaPlan (LGP) version 19.3 released on March 22, 2019, was used for planning. The dose rate at the isocenter of the GKI was about 3 Gy/minute during this period. Frame-based patients, all treated in a single fraction, had a median age of 63.5 years old, ranging from 36 to 87 years old. Frameless patients had a median age of 67 years old, ranging from 34 to 85 years old. Table 1 listed the distribution of diagnosis of frame-based and frameless cases.

**Table 1:** Summary of lesion type and fixation technique for the 57 Gamma Knife patients in this study

Type of lesion	No.	
	Frame-based	Frameless
Metastasis	8	31
Acoustic neuroma	4	
Meningioma	2	
Trigeminal nerve	1	
Arteriovenous malformation (AVM)	1	
Metastasis and surgical cavity		3
Surgical cavity		3
Vestibular schwannoma		1
Cavernous		1
Recurrent glioma		2

Data collection

For the 16 frame-based treatments, time points were recorded for the check-in, imaging completion, plan approval, and treatment start/end. The planned treatment time given by the planning software for delivering the prescribed dose was also recorded and compared with the actual treatment time or the difference between the treatment start and end time. Since this is a one-day procedure, 16 treatment plans were made and 16 single-fraction treatments were delivered.

For the frameless treatments, the check-in time on the treatment day and a few time points during the treatment were recorded. The actual treatment time in this study was defined as the time taken for the delivery of a fractionated treatment, including the initial CBCT time for localizing the treatment target(s), couch movement time from the completion of one shot to the start of the next shot, and coaching time and extra CBCT time to localize the treatment target(s) again when patient movement is out of tolerance. Therefore, the actual treatment time should be slightly longer than the planned treatment time if the patient is able to keep his/her treatment position throughout an entire treatment fraction; otherwise, the actual treatment time can be significantly longer than the planned treatment time. Another time recorded was the patient on-table time, defined as the time from the patient entering to exiting the treatment room. The patient on-table time should be longer than the actual treatment time as it also included the time for setting the patient up for CBCT. Finally, statistical data on when treatment interruptions happened and how long they last were collected. As mentioned earlier, interruption(s) may occur during a frameless treatment if the patient moves out of the preset tolerance, and as demonstrated in the later sections, they were the main contributors to the difference between the actual and planned treatment time.

Among the 41 frameless cases, 47 treatment plans were made and 91 treatment fractions were delivered. Twenty of the frameless patients were treated in a single fraction. Of the 21 fractionated frameless treatments, 17 cases were treated in three fractions and four cases in five fractions. Table 2 lists the number of cases for each of the three fractionation schemes. Most three-fraction patients were treated with one treatment plan except two, who were treated with two treatment plans and another two with three treatment plans. The reason for more than one plan for an individual patient was that some of the targets got three fractions and the rest received only one fraction. When there were two or more plans for a patient, the planned treatment time was also balanced among different plans.

**Table 2:** Number of cases for each of the 3 fractionation schemes for frameless treatments.

Number of fractions	Number of cases
1	20
3	17
5	4

The time spent from the completion of one patient to the initiation of the next patient was recorded for the whole treatment day, regardless of the type of frame-based or frameless treatments.

Workflow analysis for frame-based treatment

The workflow for frame-based treatment consists of all six steps. The analysis was focused on the time spent for each step as well as the total time (i.e., patient in-clinic time) of the whole process. The time spent for each step, as well as the patient's in-clinic time, was calculated from the recorded time points. In addition, the idle time between steps was also calculated and analyzed to find its correlation with the total time of the whole process.

Workflow analysis for frameless treatment

Since steps (1) to (5) of the workflow for frameless treatment may happen prior to the treatment day, the analysis was focused on the comparison of the actual treatment time and planned treatment time and the effects of major treatment interruptions, defined as those resulting in a couch retraction and a significant treatment delay by at least 10 minutes. The histogram of when major interruptions happened was first constructed, from which the mean and standard deviations of the event time were derived. The major interruptions lead to an increased treatment time. To analyze this effect, the deviation of the actual treatment time from the planned treatment time was calculated as the difference between these two times. We then plotted the time difference versus the planned treatment time and ran a piecewise linear regression that allows multiple linear segments of fitted value for different ranges of a covariate [9]. The purpose of this analysis was threefold: (1) to determine if the time difference was correlated with the length of planned treatment time; (2) if those two times were correlated, was the correlation linear, or (3) piece-wise linear, i.e., there exist one or more break points so that the data fit a linear regression within each segment. The breakpoints were detected when the differences in the slopes of multiple segments obtained statistical significance. Treatments with breaks were excluded from data fitting and a two-sided P-value <0.05 was considered statistically significant. The results from this analysis shed light on, statistically, how long a patient could maintain the treatment position without major breaks that would significantly increase the actual treatment time.

## Results

Frame-based cases

**Figure 1:**
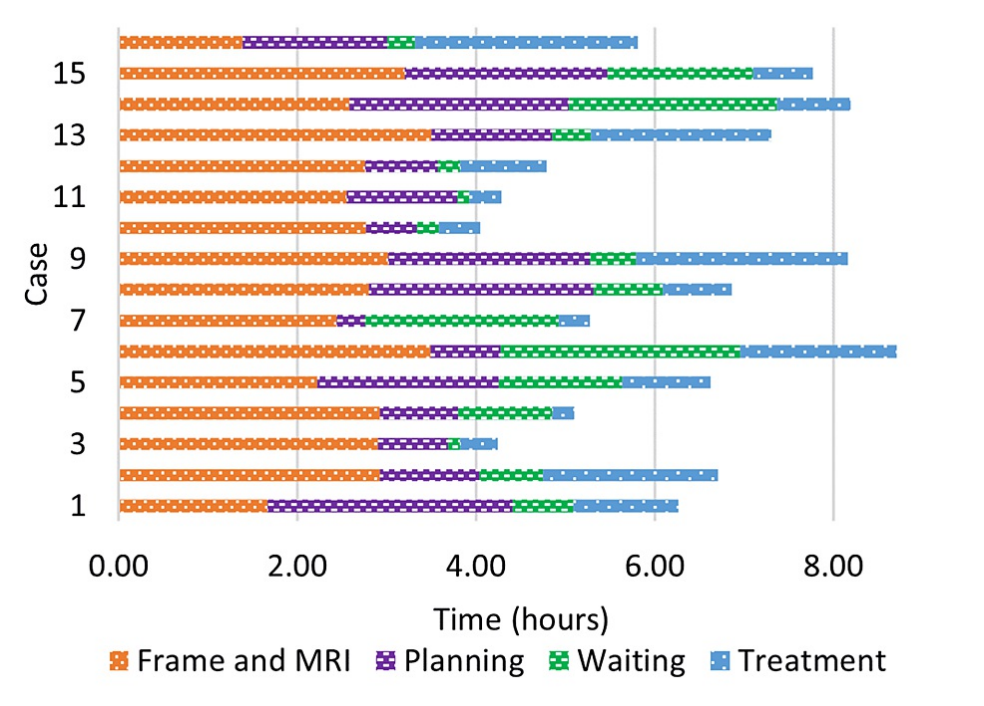
The time spent for completing each step on the treatment day for each frame-based treatment

For frame-based cases, the time taken for completing each step of the treatment for each patient is shown in Figure 1. The median time from patient check-in to MRI completion was 2.7 hours (ranging from 1.4 to 3.5 hours) and the time from MRI completion to plan approval was 1.5 hours (ranging from 0.3 to 2.7 hours). After plan approval, the time to the initiation of treatment varied from 8 minutes to 2.7 hours with an average of one hour. The planned treatment time varied from 15 minutes to 2.5 hours with an average of 1.1 hours. The total time in the clinic before treatment completion varied from 4 to 8.7 hours with an average of 6.25 hours. For frame-based treatments, we have established a six-hour goal at our clinic for the time from check-in to treatment completion. In this study, seven out of 16 (44%) cases were completed within this time frame.

Frameless cases

**Figure 2:**
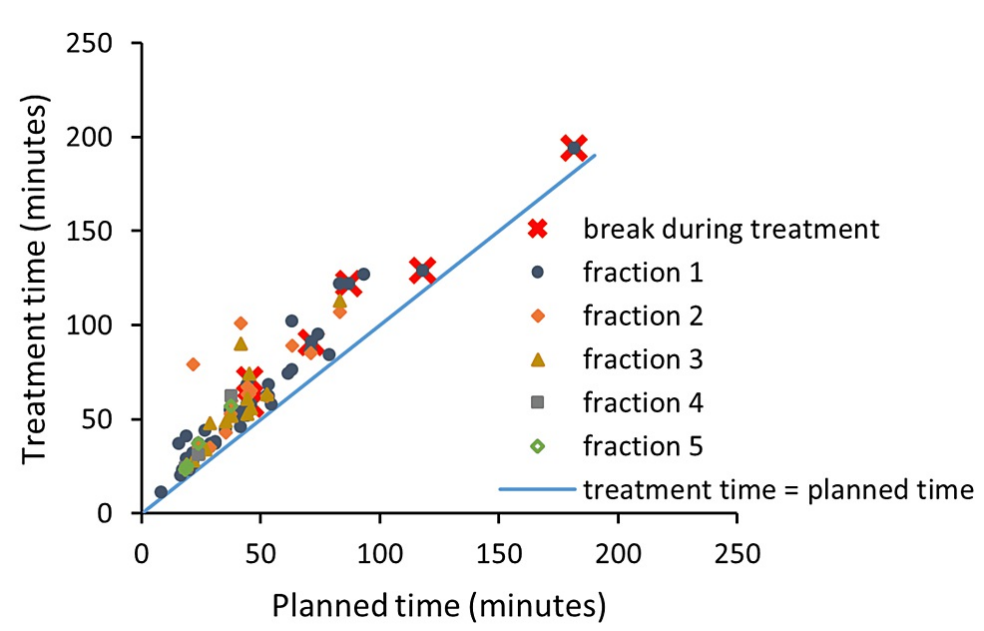
The actual treatment time versus the planned treatment time for frameless cases Data points with cross signs indicate treatments with planned or unplanned breaks.

Table 3 lists the distribution of the planned treatment time for the 47 frameless plans, among which 35 (74.5%) plans were less than one hour and 30 (63.8%) plans were between 10 and 50 minutes. Figure 2 illustrates the actual treatment time versus the planned treatment time for frameless cases. In all cases, the actual treatment time was longer than the planned time by 3 to 59 minutes (median 11 minutes). The actual treatment time includes the pre-treatment CBCT, which takes approximately 4 minutes and is not reflected in the planned treatment time.

**Table 3:** Distribution of the planned treatment time for the 47 frameless plans

Planned treatment time (minutes)	Number of plans
< 10	1
10-20	8
20-30	8
30-40	6
40-50	8
50-60	4
60-70	3
70-80	4
80-90	2
>90	3
Total	47

A subgroup analysis was performed for the 50 treatments, of which the actual treatment time was 10 minutes longer than the planned treatment time. Major treatment interruptions were more commonly encountered in patients with a planned treatment time over 40 minutes, which accounts for 64% of those 50 treatments. In comparison, only ~44% of the total 91 treatments had a planned treatment time of over 40 minutes. Most (88%) of the cases with at least one major treatment interruption were caused by patients not being able to hold the treatment position and moving out of the tolerance. The other 12% were due to machine errors that led to a frozen system and required reinitialization to clear. It was later diagnosed that the router responsible for the communication between the treatment console and the primary GK computer was defective. Three patients required breaks, in which the patient left the treatment room for more than 10 minutes before resuming. There were planned breaks in advance for two patients with planned treatment times of 118 and 181 minutes, which were not counted toward the actual treatment time.

In some cases, treatment interruption required one or more extra CBCTs other than the initial CBCT to align patients before treatment could be resumed. The maximum number of extra CBCTs required in one treatment fraction was 4. Table 4 listed the number of extra CBCT and their frequency of occurrence in the 91 frameless treatments.

**Table 4:** Number of extra CBCTs required to align the patients for treatments and their frequency of occurrence in the 91 frameless cases CBCT: cone-beam computed tomography

Number of extra CBCTs	Frequency of occurrence
0	58
1	24
2	6
3	1
4	2

**Figure 3:**
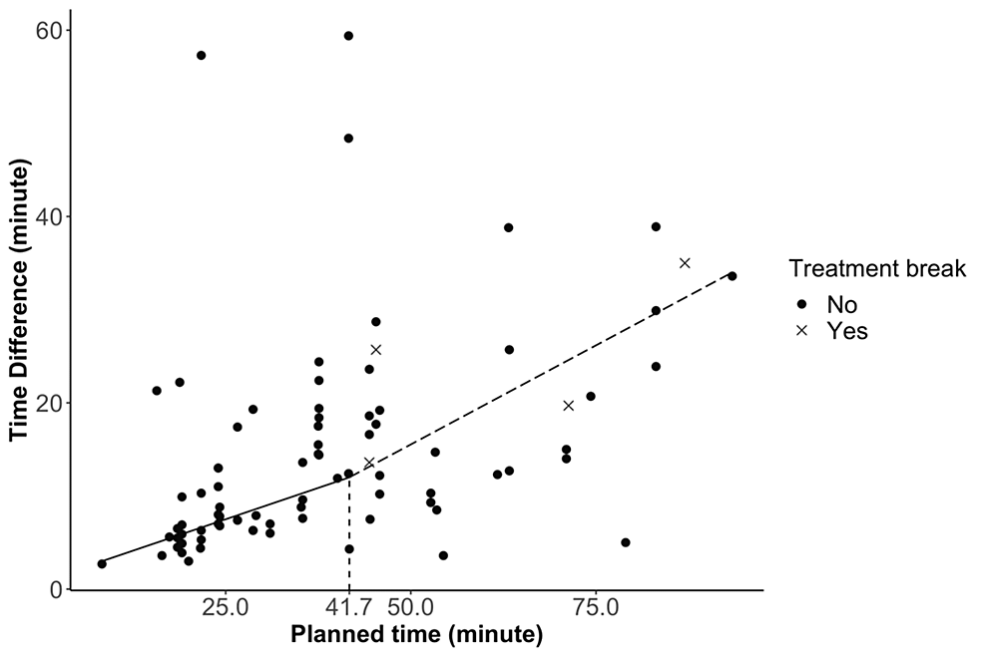
The time difference between the planned and actual treatment time versus the planned treatment time Solid and dashed lines are the two fitted linear regression lines for the two data segments separated by the planned treatment time of 41.7 minutes.

Figure 3 shows the results from the piecewise linear regression, where the deviation from the treatment time is the best fit with two segments separated by the 41.7-minute breakpoint. The solid and dashed lines are the two fitted regression lines for the two data segments: segment I with a planned treatment time of less than 41.7 minutes and segment II with greater than 41.7 minutes. The slope of the linear fit to the data in segment II is significantly higher than that in segment I (P=0.01), which could be interpreted as when the planned treatment time is longer than 41.7 minutes, it is more difficult for patients to keep their treatment positions within the tolerance. Therefore, the extra time required to complete the treatment increases more rapidly with the increase of planned treatment time for treatment longer than 41.7 minutes.

Transition time between patients

**Figure 4:**
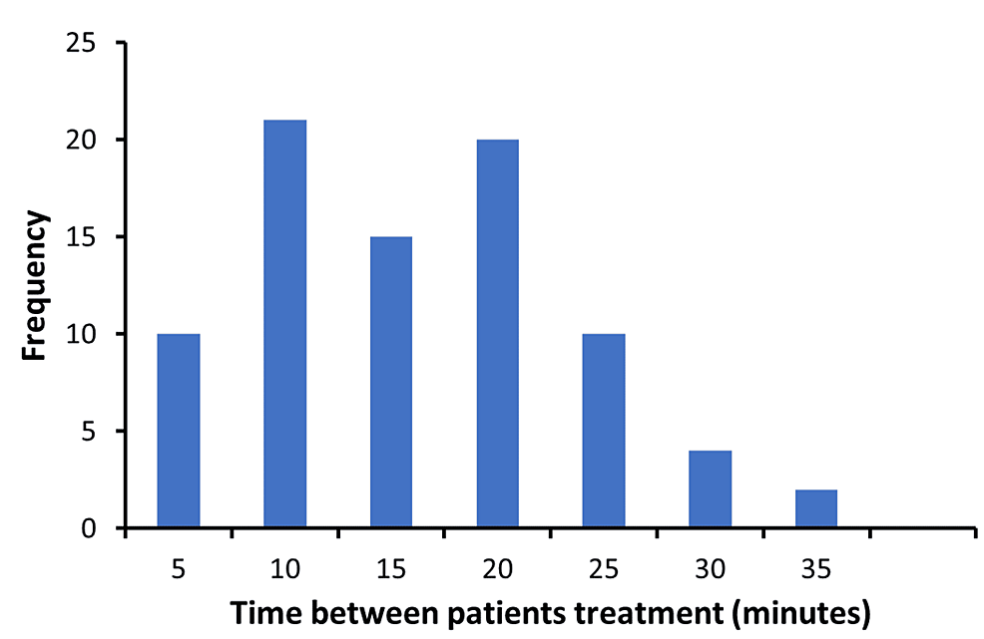
Frequency of time intervals needed to switch patients between treatments

The time taken from the completion of one treatment to the initiation of the next treatment, defined as the time the previous patient left the treatment room to the time the subsequent patient entered the treatment room is listed in Figure 4. This transition time varied from less than 5 minutes to 35 minutes with a mean of 15 minutes. Note that the time interval between two patients was not included if they were scheduled more than 40 minutes apart.

## Discussion

Workflow optimization is critical to the resource management and patient experience in a busy clinic. Reviewing our experience at a large center incorporating both frame-based and frameless GK radiosurgery, we found that treatment times varied quite significantly amongst patients.

One major obstacle to meeting this 6-hour goal for completing frame-based treatment is the time spent on frame placement and MRI imaging. As shown in Figure 1, it took an average of 2.7 hours to complete these two steps. We have attempted to reduce this time by creating a block schedule for GK cases with appropriate staffing in order to expedite pre-medications/paperwork, ensure neurosurgeon availability, and better coordinate time-slots with Radiology. Of the nine patients with in-clinic time greater than 6 hours, six cases had a planned treatment time longer than one hour. The planned treatment time was largely determined by factors including the size, shape, location, and number of targets, most of which are not completely determined until after the MRI imaging on the treatment day. Since a longer treatment time always leads to a longer in-clinic time, it is therefore extremely challenging to control all the patient's in-clinic time to be within 6 hours.

Even though the time for treatment delivery (step 6) is hard to control, the patient's in-clinic time for framed treatments can still be shortened by optimizing the workflow of the other five steps. For example, it took an average of 1.5 hours to develop and approve a plan for frame-based treatments. This time can potentially be reduced with the most recent GK software updates. It was a well-known limitation in the past that the GK planning software did not accept DICOM (Digital Imaging and Communications in Medicine) image sets with non-square pixels for planning, and therefore no planning activities could be taken beforehand. The software was upgraded in September 2019 to recognize diagnostic MRI images even if the DICOM image sets contained non-square pixels. Although the pre-planned treatment plan still needed to be transferred to the localized CBCT or MRI images and finalized on the day of treatment, the time required is much less than that for planning from scratch. We plan to implement pre-planning in our frame-based workflow to determine if the planning time can be shortened on the treatment day.

For frameless treatments, the major uncertainty is the patients’ ability to maintain their head position throughout each treatment fraction. When the patient moves out of tolerance, the treatment is held and the patient needed to be coached back to the proper position. If the patient is not able to return to the treatment position within 30 seconds, another CBCT is required, which prolongs the treatment time. For example, in one extreme case of this study, the planned treatment time was 42 minutes, but it took 101 minutes to complete the treatment, as three additional CBCT scans were required to re-localize the patient.

The analysis (Figure 3) showed that when the planned treatment time was longer than 41.7 minutes, the patient was more likely to have difficulty maintaining position and more time was needed to complete the treatment. Therefore, to reduce the actual treatment time for cases with a long, planned treatment time, it might be a good practice to schedule one or two planned breaks during the treatment. The advantage of this practice was actually evidenced in the data we collected for this study. As shown in Figure 2, for the two longest treatments, the extra time it took to complete the planned treatments was actually less than that of many cases with a planned treatment time of under 42 minutes. We are aware that it is difficult to make a definitive conclusion base on such a small sample size. It would be interesting to test this hypothesis in future practice. Another option to minimize the actual treatment time is to fractionate the treatment to decrease the length of any individual treatment on a given day. This could be done by either treating different lesions on different days as we already did for some of the patients or by increasing the fractionation size, e.g., from three to five fractions to reduce the dose that needs to be delivered on a single day.

As shown in Figure 4, the recorded transient time varied significantly from 5 to 35 minutes. This transient time can be used as an indicator to quantify how efficient the therapist was in switching patients for treatments. In theory, the therapist could clean the treatment couch and have the room ready for the next patient in a few minutes. However, sometimes, this transient process was significantly slowed down due to unexpected extra work, e.g., documentation for previous treatment, patient’s last-minute request to use the restroom, or additional medications before entering the treatment room. This delay can be minimized with proactive scheduling. For example, we can start preparing the next patient for treatment 5-10 minutes before, instead of waiting until the completion of the current treatment. In addition, the nurse should check the patient's conditions periodically and administer the necessary medication when there is a need. Finally, when the patient's treatment is behind schedule, non-urgent documentation that does not need to be completed immediately after the treatment can be completed at the end of the day when all treatments are finished.

The workflow efficiency also depends on the experience and expertise of the medical staff. In general, experience and expertise can be quantified by the procedure count and the training received but were not measured because all cases in this study were performed by the same medical staff. Even though these data were not available, the authors can state, based on our clinical observations, that the most significant experience and expertise that might impact workflow efficiency is the therapist’s ability to guide the patient back to the correct position when the monitored patient motion is above the preset threshold. As pointed out early, a major interrupt leads to (1) treatment pause, (2) re-CBCT, and (3) moving the patient into the treatment position again. The number of these costly operations can be reduced if an experienced therapist can better guide the patient back to the correct treatment position in time.

The authors would like to point out that the purpose of this study was to analyze and optimize the workflow of GK frame-based and frameless treatments at our institution. How patients were selected for frame-based or frameless treatment and how the fractionation scheme was determined is a clinical decision and is beyond the scope of this study. We are analyzing the outcomes of patients treated with frame-based and frameless approaches and the results will be published in the near future. The limitation of this study is that this is a retrospective analysis based on the experience of a single radiation oncology department.

## Conclusions

Improving the efficiency of the GK workflow requires the collaborative effort of the entire GK team. For frame-based patients, most of the treatment day is spent on frame placement/MRI completion/treatment planning. With the new GK pre-planning feature, some of the work can be performed beforehand, making the scheduling more predictable and therefore the workflow more efficient. For frameless patients, the major challenge is the patient being unable to hold the position throughout the entire treatment. Particularly, a planned treatment time greater than 41.7 minutes is associated with a greater chance of treatment interruptions due to intra-fractional motion. The efficiency can therefore be improved with a careful selection of fractionation schemes so that the planned treatment time is less than 41.7 minutes. For those cases requiring a longer treatment time, planned treatment breaks may prevent unexpected interruptions, which usually take a longer time to clear.
